# Restraining Akt1 Phosphorylation Attenuates the Repair of Radiation-Induced DNA Double-Strand Breaks and Reduces the Survival of Irradiated Cancer Cells

**DOI:** 10.3390/ijms19082233

**Published:** 2018-07-31

**Authors:** Klaudia Szymonowicz, Sebastian Oeck, Adam Krysztofiak, Jansje van der Linden, George Iliakis, Verena Jendrossek

**Affiliations:** 1 Institute of Cell Biology (Cancer Research), University of Duisburg-Essen, University Hospital Essen Virchowstrasse 173, 45147 Essen, Germany; klaudia.szymonowicz@uk-essen.de (K.S.); sebastian.oeck@uk-essen.de (S.O.); adam.krysztofiak@uk-essen.de (A.K.); janettevanderlinden@hotmail.com (J.v.d.L.); 2 Institute of Medical Radiation Biology, University of Duisburg-Essen, University Hospital Essen, Virchowstrasse 171, 45147 Essen, Germany; georg.iliakis@uk-essen.de; 3 Department of Therapeutic Radiology, Yale University School of Medicine, 15 York Street, New Haven, CT 06520, USA

**Keywords:** Akt, protein kinase B, Akt-phosphorylation, radiosensitivity, T308A, S473A, DNA-PK, MERIT40, radiation, DNA damage, DSB repair

## Abstract

The survival kinase protein kinase B (Akt) participates in the regulation of essential subcellular processes, e.g., proliferation, growth, survival, and apoptosis, and has a documented role in promoting resistance against genotoxic stress including radiotherapy, presumably by influencing the DNA damage response and DNA double-strand break (DSB) repair. However, its exact role in DSB repair requires further elucidation. We used a genetic approach to explore the consequences of impaired phosphorylation of Akt1 at one or both of its key phosphorylation sites, Threonine 308 (T308) or Serine 473 (S473), on DSB repair and radiosensitivity to killing. Therefore, we overexpressed either the respective single or the double phosphorylation-deficient mutants (Akt1-T308A, Akt1-S473A, or Akt1-T308A/S473A) in TRAMPC1 murine prostate cancer cells (TrC1) and measured the DSB repair kinetics and clonogenic cell survival upon irradiation. Only the expression of the Akt1-T308A/S473A induced a significant delay in the kinetics of DSB repair in irradiated TrC1 as determined by the γH2A.X (H2A histone family, member X) assay and the neutral comet assay, respectively. Moreover, Akt1-T308A/S473A-expressing cells were characterized by increased radiosensitivity compared to Akt1-WT (wild type)-expressing cells in long-term colony formation assays. Our data reveal that Akt1’s activation state is important for the cellular radiation response, presumably by modulating the phosphorylation of effector proteins involved in the regulation of DSB repair.

## 1. Introduction

The PI3K (phosphatidylinositol-3-kinase)/Akt (protein kinase B) pathway is aberrantly activated in many types of cancer, and associated with resistance to cancer therapy and poor prognosis [1,2,3]. The serine/threonine kinase Akt impacts several different cellular processes, e.g., protein synthesis, cell metabolism, cell proliferation, apoptosis, and cell survival under stress conditions, by targeting various effector proteins (reviewed by [4,5]). The activation of Akt is initiated by phosphorylation at two sites, namely T308 (Threonine308) by phosphoinositide-dependent kinase-1 (PDK1) and S473 (Serine473) by PDK2 family members such as mTORC2 (mammalian target of rapamycin complex 2) or DNA-PK (DNA-dependent protein kinase), respectively [6,7,8] (reviewed by References [4,5]). The initial phosphorylation at T308 depends on recruitment of Akt to the cellular membrane, a process that is initiated by binding of Akt’s PH domain (pleckstrin homology domain) to phosphatidylinositol-(3,4,5)-trisphosphate (PIP_3_) at cellular membranes. However, the aberrant activation of Akt can also occur as a result of gain-of-function mutations in Akt or the upstream regulatory kinase PI3K, by gene amplification of one of the three Akt isoforms, or by loss-of-function of the tumor suppressor PTEN (phosphatase and tensin homolog) respectively; the latter negatively regulates PIP_3_ production that is indispensable for Akt membrane recruitment and phosphorylation (reviewed by References [4,5]). Akt-activating mutations are frequently observed in cancer cells and not only increase cell proliferation and cell survival but are also suspected to alter the DNA damage response and to improve the repair of DNA damage [6,7,9,10,11].

Herein, there is increasing evidence that the genetic or stress-induced activation of Akt impacts the repair of DNA double-strand breaks (DSB) by phosphorylating downstream effector proteins that are involved in the regulation or execution of one of the two main pathways of DSB (double strand break) repair: non-homologous end joining (NHEJ) and homologous recombination repair (HRR) [12,13]. These included, for example, XLF (XRCC4-like factor) and UBE2S (ubiquitin-conjugating enzyme E2 S) as Akt target proteins connected to NHEJ, as well as MERIT40 (mediator of Rap80 Interactions and Targeting 40 kDa), and EMSY (BRCA2-interacting transcriptional repressor) with suggested functions in HRR [9,10,11,14,15]; reviewed by Reference [5].

We recently demonstrated that the catalytic subunit of DNA-PK (DNA-PKcs) acts as a direct upstream kinase of Akt1-S473, at least in vitro. Moreover, we revealed that the overexpression of the clinically relevant mutant Akt1-E17K with an improved membrane recruitment, or of an artificial constitutively active phosphorylation-mimicking Akt1 mutant (T308D/S473D), significantly accelerated the repair of radiation-induced DSB and increased the radioresistance of TrC1 when compared to Akt1-WT overexpressing cells [7]. However, the importance of phosphorylation at T308, S473 or both in the above processes remained elusive. We speculated that impaired Akt1 activation might impact DNA repair processes and cell survival upon irradiation by precluding phosphorylation of downstream proteins with a reported role in DSB repair.

Here, we further explored the importance of Akt’s-phosphorylation at S473 and T308 in the regulation of the DSB repair and radiosensitivity. For this, we analyzed the role of DNA-PKcs as an upstream kinase in radiation-induced Akt-S473 phosphorylation in intact cells using DNA-PK-proficient and DNA-PK-deficient human glioblastoma cell lines [16]. Moreover, we established murine TrC1 prostate cancer cells stably overexpressing phosphorylation-deficient Akt1 mutants including Akt1-T308A (Akt1-TA), -S473A (Akt1-SA) and -T308A/S473A (Akt1-TASA) and determined the effects of this overexpression on DNA DSB repair as well as on its survival upon exposure to ionizing radiation (IR).

## 2. Results

### 2.1. DNA-PKcs Participates in Radiation-Induced Akt S473 Phosphorylation in Glioblastoma Cells

**Figure 1:**
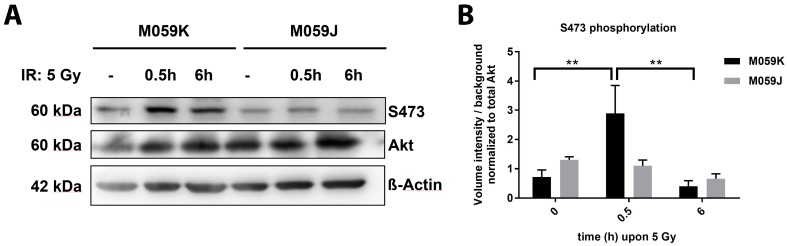
The DNA-PKcs phosphorylates Akt-S473 in cancer cells upon irradiation. Human glioblastoma cell lines M059J (DNA-PK deficient) and M059K (DNA-PK proficient) were exposed to irradiation (IR) with 5 Gray (Gy). (**A**) The phosphorylation (S473) and expression status of Akt was determined by western blot analysis 0.5 and 6 h upon IR and in non-irradiated (-) cells. (**B**) The quantification of pS473 western blots of 3 independent experiments shows the volume intensity normalized to the background. The volume intensity of phosphorylated Akt-S473 was normalized to the volume intensity of the total amount of Akt. The bars show the mean values ± SD of 3 independent experiments ** *p* < 0.01 ANOVA test with Tukey correction.

Our earlier work revealed that DNA-PKcs phosphorylates Akt1 at S473 but not vice-versa in the presence of damaged DNA, at least in a protein-based in vitro assay [7]. To explore if DNA-PKcs might also execute radiation-induced Akt-S473 phosphorylation in intact cancer cells, we analyzed the time-dependent phosphorylation of Akt-S473 in irradiated DNA-PKcs-deficient (M059J) and DNA-PKcs-proficient (M059K) human glioblastoma cell lines (Figure 1; Figure S1A). Indeed, we observed a transient but significant increase in phosphorylation of Akt-S473 in the DNA-PKcs-proficient cell line M059K upon IR with 5 Gray (Gy) that was not detected in the DNA-PKcs-deficient cell line M059J (Figure 1A,B). Here, the level of Akt-S473 phosphorylation remained constant during the observation period. Similar observations were made when Akt-S473 phosphorylation was analyzed upon irradiation with 3 Gy (Figure S1A,B). Furthermore, we compared the kinetics in the repair of radiation-induced DSB in both cell lines upon exposure to a single dose of 3 Gy and were able to corroborate the dysfunction of DSB repair in DNA-PKcs-deficient M059J cells [17] (Figure S1C).

### 2.2. Phosphorylation-Deficient Mutants Akt1-SA and -TASA Enhance the Radiosensitivity of TrC1 Prostate Cancer Cells

**Figure 2:**
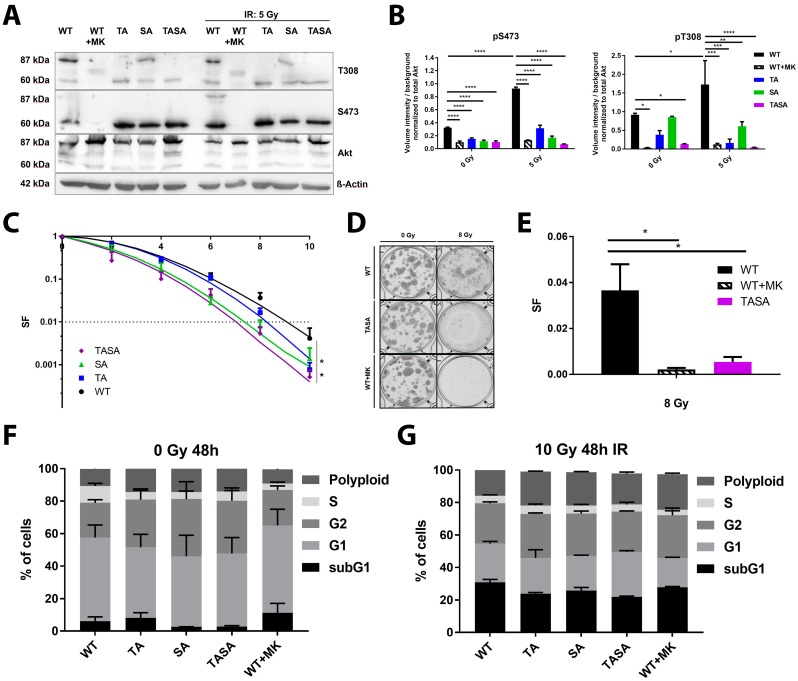
Expression of phosphorylation-deficient Akt1 mutants reduced cancer cell radiosensitivity. TrC1 were exposed to irradiation with 5 Gy. (**A**) The phosphorylation status (S473, T308) of the Akt1 mutants at 0.5 h after irradiation depicted by western blot analysis. Lower bands (60 kDa) show endogenous Akt; upper bands (87 kDa) depict eGFP-fused Akt1-mutants. (**B**) The quantification of pS473 and pT308 western blots of 3 independent experiments shows the volume intensity normalized to the background. The volume intensity of phosphorylated Akt was normalized to the volume intensity of total amount of Akt. (**C**,**D**) Long-term survival (survival fraction, SF) altered by Akt1 mutants upon IR (0–10 Gy). Akt1-TASA showed significantly reduced survival upon IR. Pictures depict a standard 6-well cell culture plate. (**E**) Long-term survival in Akt1-WT expressing cells treated with 4 µM MK-2206 for 16 h before IR (WT + MK) compared to the effect evoked by Akt1-WT and Akt1-TASA expression without additional treatment. Data represent SF upon 8 Gy. * *p* < 0.05, ** *p* < 0.01, *** *p* < 0.001, **** *p* < 0.0001; ANOVA test with Tukey correction. (**F**,**G**) Quantification of cell cycle distribution in non-irradiated (**F**) and with 10 Gy irradiated (**G**) Akt1-WT, Akt1-TA, Akt1-SA, Akt1-TASA expressing cells and Akt1-WT expressing cells treated with an MK-2206 inhibitor (4 µM; 16 h incubation; WT + MK) were analyzed by flow cytometry after 48 h incubation. Data show mean values from 3 independent experiments.

Our previous data also indicated that the activation-associated mutations of Akt accelerate DSB repair and improve the survival of irradiated cancer cells, suggesting that Akt-activation might be crucial for its repair-promoting effects [7]. To gain more insight into the importance of Akt-phosphorylation at S473 and T308 for its role in the cellular radiation response, we generated TrC1 stably expressing phosphorylation-deficient eGFP-fused Akt1 mutants Akt1-TA, Akt1-SA, and Akt1-TASA by using retroviral gene transfer (Figure 2A,B). For a better comparability of data obtained in the generated cell lines, we adjusted the expression level of Akt1-eGFP fusion proteins in all generated cell lines by cell sorting based on the eGFP-intensity ensuring that the GFP-fused Akt-variants were expressed at largely increased levels compared to the endogenous protein (Figure 2A). We also confirmed the lack of phosphorylation of the overexpressed double phosphorylation-deficient Akt1-TASA-eGFP fusion protein (87 kDa) whereas the 60 kDa endogenous Akt protein was still phosphorylated at S473 and T308 (Figure 2A,B).

The exposure of Akt1-WT overexpressing TrC1 to irradiation with 5 Gy increased phosphorylation of both, endogenous Akt and the overexpressed Akt1-WT protein, at T308 and S473. Instead, the pre-treatment of Akt1-WT overexpressing TrC1 for 16 h with 4 µM of the Akt-inhibitor MK-2206 led to the complete abrogation of basal and radiation-induced Akt1-T308 and Akt1-S473 phosphorylation of both, endogenous Akt and overexpressed Akt1-WT (Figure 2A,B; quantification of endogenous phosphorylated Akt is shown in Figure S2D).

Of note, we observed increased phosphorylation of the overexpressed Akt1-SA mutant at T308 under basal conditions and upon irradiation with 5 Gy, whereas we could not detect any phosphorylation of the phosphorylation-deficient Akt1 mutants at Akt1-S473, even in the single T308 phosphorylation-deficient Akt1-TA mutant, neither under basal conditions nor upon irradiation (Figure 2A,B). These results suggest that T308 might be essential for S473 phosphorylation and cannot occur in cells with impaired T308-phosphorylation. 

To evaluate the suspected failure of the phosphorylation-deficient mutants to phosphorylate known downstream targets of Akt1, we next compared the ability of Akt1-WT and Akt1-TASA expressing cells to phosphorylate the FOXO1 (forkhead-box-protein O1) transcription factor, a documented target of Akt important to Akt´s role in apoptosis regulation (reviewed by Reference [18]). As expected, we observed reduced basal phosphorylation of FOXO1 in Akt1-TASA overexpressing cells. Similarly, phosphorylation of FOXO1 was also reduced in Akt1-WT expressing cells treated with MK-2206 (Figure S2A). 

Next, we analyzed if the overexpression of the phosphorylation-deficient Akt mutants would alter the radiosensitivity of TrC1. For this, we compared the long-term survival upon IR in all cell lines using standard colony formation assays. These investigations revealed that overexpression of phosphorylation-deficient Akt1–SA and Akt1–TASA mutants enhanced the radiosensitivity of TrC1 when compared to Akt1-WT expressing cells (Figure 2C). A similar effect could be achieved by treating Akt1-WT expressing cells with the Akt-inhibitor MK-2206 (WT + MK; Figure 2D,E). Instead, we detected only a minor effect of Akt1-TA overexpression on the survival of irradiated TrC1 in comparison to Akt1-WT (Figure 2C).

To exclude a potential influence of the phosphorylation-deficient mutants on the cell cycle, we also compared the cell cycle distribution in our cell lines. However, we did not observe any significant differences in the cell cycle distribution between all Akt1-mutants upon exposure to IR (Figure 2F,G; Figure S2B,C).

### 2.3. Phosphorylation Status of Akt Is Not Crucial for Nuclear Localization and Its Translocation Upon IR

Our previous data indicated the increased radioresistance of cells with the overexpression of the activation-associated Akt1 mutants Akt1-TDSD and Akt1-E17K. Moreover, increased radioresistance of Akt1-TDSD and Akt1-E17K was associated with enhanced nuclear localization upon IR and accelerated DSB repair [7]. However, it remained unclear if phosphorylation of Akt is required for its nuclear translocation and suspected action on nuclear target proteins, particularly upon DNA damage. Here, we used eGFP-fused Akt1-mutants to reveal potential differences in the nuclear amount of Akt between the phosphorylation-proficient and phosphorylation-deficient Akt1-mutants under basal conditions and upon irradiation. 

**Figure 3:**
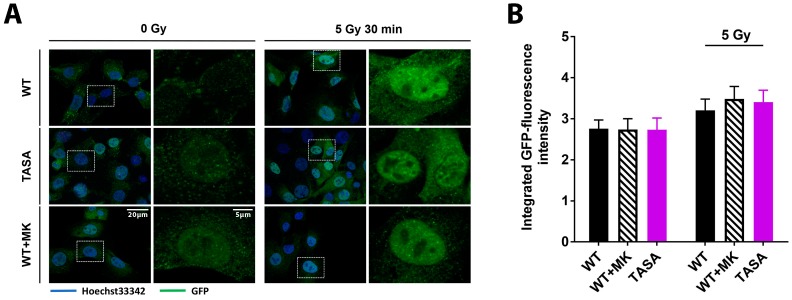
The basal Akt phosphorylation is not required for its nuclear localization**.** TrC1 Akt1-TASA, Akt1-WT and Akt1-WT expressing cells pretreated with solvent or 4 µM MK-2206 (2 h before IR) were exposed to IR with 0 Gy or 5 Gy. 30 min after the irradiation cells were fixed in 3% paraformaldehyde, permeabilized with 0.2% Triton X-100 in phosphate-buffered saline (PBS), and subjected to immunofluorescence analysis. (**A**) Representative photomicrographs showing subcellular localization of the eGFP-coupled Akt1 variants Akt1-TASA and Akt1-WT upon the indicated treatments. The overview pictures were done using 63-fold magnification. Detailed pictures were obtained using 63-fold magnification. (**B**) Quantification of the integrated eGFP-intensity in nuclei of TrC1 expressing Akt1-TASA and Akt1-WT mutants with and without pre-treatment with the MK-2206 inhibitor. Quantification involves the analysis of 50 cells per condition and was performed by a CellProfiler software [19]. Data show means ± SD from 3 independent experiments; ANOVA test with Tukey correction and showed no significant differences.

Interestingly, TrC1 overexpressing either Akt1-TASA or Akt1-WT showed similar basal levels of nuclear Akt as depicted by the immunocytochemistry and quantification of the integrated eGFP fluorescence intensity in the nuclei using the CellProfiler software (Figure 3A,B) [19]. In line with these findings, the treatment of Akt1-WT expressing TrC1 with the allosteric Akt-inhibitor MK-2206 did not alter the basal nuclear Akt-levels suggesting that the phosphorylation of Akt1 at T308 and S473 is not required for Akt´s nuclear localization. Finally, exposure to IR induced a comparable slight but insignificant increase in nuclear Akt1-eGFP in all tested Akt1-mutants, arguing against a pronounced effect of Akt´s phosphorylation state on its subcellular localization (Figure 3A,B).

### 2.4. Overexpression of the Phosphorylation-Deficient Akt1-TASA Mutant Delays the Kinetics of DNA Repair Upon Irradiation, Potentially via Decreased Phosphorylation of Effector Proteins with an Impact on DSB Repair

**Figure 4:**
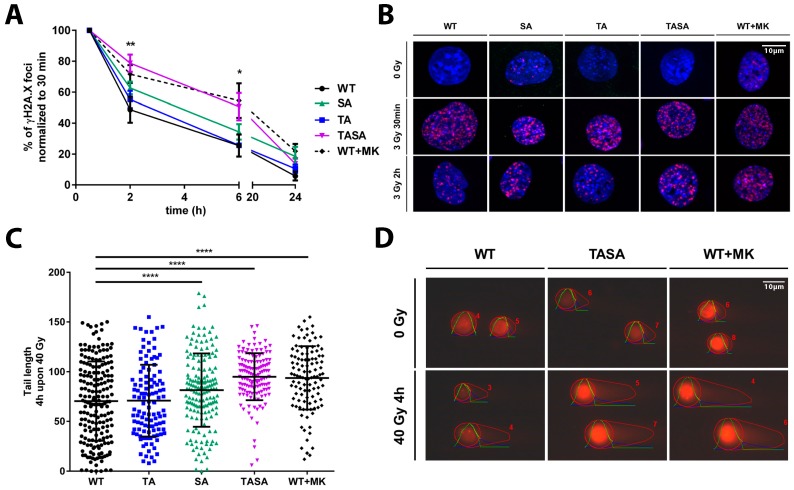
The genetic or pharmacologic inhibition of Akt1-phosphorylation affects DNA repair upon IR. TrC1 stably overexpressing Akt1-WT, pre-treated for 2 h with 0 or 4 µM MK-2206, or the phosphorylation-deficient Akt1-TA, -SA or -TASA mutants were exposed to irradiation with 0 Gy, 3 Gy (**A,B**) or 40 Gy (**C,D**) as indicated. (**A**,**B**) Cells were fixed in 3% para-formaldehyde (PFA), permeabilized with 0.2% Triton X-100 in phosphate-buffered saline (PBS) at distinct time points between 0 h and 24 h upon irradiation with 3 Gy, and stained with Hoechst33342, to visualize the nuclei (blue), and γH2A.X (magenta), to visualize sites of DNA DSB. (**A**) The number of γH2A.X foci at 2–24 h after irradiation with 3 Gy using the Focinator v 2.2. software [20] was normalized to the number of foci detected at 0.5 h time point. (**C**,**D**) Cells were processed by applying neutral comet assay to quantify the amount of damaged DNA in the form of DSB at a fixed time-point. The quantification was performed by the OpenComet software and depicts the comet tail length of each indicated Akt1 mutant 4 h upon 40 Gy. (**A**,**C**) Data show means ± SD from 3 independent experiments with 50 analyzed nuclei per trial and condition. * *p* < 0.05, ** *p* < 0.01, *** *p* < 0.001, **** *p* < 0.0001; ANOVA test with Tukey correction. (**B**,**D**) Data show representative pictures out of 3 independent experiments.

Thus far, our data indicated that the overexpression of the phosphorylation-deficient Akt1 mutants Akt1-SA or Akt1-TASA increased the radiosensitivity of TrC1 when compared to Akt1-WT overexpressing TrC1 (Figure 2C–E). Since our earlier work revealed an association of increased radioresistance of TrC1 overexpressing activation-associated Akt1-mutants with alterations in the kinetics of radiation-induced DSB repair, we hypothesized that the overexpression of the phosphorylation-deficient Akt1 mutants might affect DSB repair. We, therefore, compared the effects of the genetic or pharmacologic inhibition of Akt1-phosphorylation at T308 and S473 on the kinetics of radiation-induced DSB repair in TrC1. The overexpression of the Akt1-TASA mutant with impaired phosphorylation at T308 and S473, as well as the treatment of TrC1 overexpressing Akt1-WT with the Akt-inhibitor MK-2206 led to a significant deceleration of DSB repair upon irradiation as determined by the γH2A.X assay (Figure 4A,B). Instead, the resolution of γH2A.X was only slightly slower in Akt1-SA overexpressing cells without reaching significant levels. 

To corroborate these observations, we additionally evaluated the amount of DSB by using the neutral comet assay. Again, the overexpression of Akt1-TASA, as well as pre-treatment of Akt1-WT overexpressing TrC1 with the Akt-inhibitor MK-2206, led to a significant increase in residual DSB at 4h after irradiation when compared to Akt1-WT overexpressing cells (Figure 4C,D). Moreover, the overexpression of Akt1-SA also significantly reduced the DSB repair compared to Akt1-WT or Akt1-TA overexpressing TrC1 in this assay (Figure 4C,D).

These findings suggested that the reduced activation of Akt1 and the resulting failure of Akt1 to phosphorylate downstream effector proteins might contribute to the observed negative effects of the phosphorylation-deficient Akt mutants on DSB repair and increased cellular radiosensitivity. 

**Figure 5:**
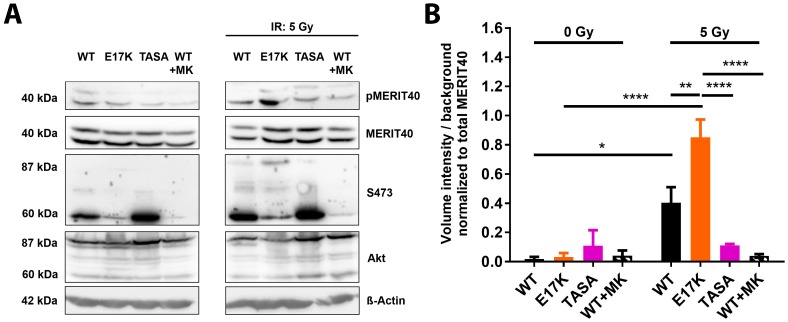
The Akt’s activation state impacts the phosphorylation of the HRR-associated Akt-target protein MERIT40. (**A**) TrC1 stably expressing the constitutively active Akt1-E17K, phosphorylation-deficient Akt1-TASA or Akt1-WT were exposed to irradiation with 5 Gy. Akt1-WT expressing TrC1 were additionally treated with 4 µM of MK-2206 2 h prior to IR. Phosphorylation status (S473) of the Akt1 mutants, as well as the expression and phosphorylation status of the assumed Akt-target protein MERIT40, at 0.5 h after irradiation depicted by western blot analysis. For S473 and Akt: lower bands (60 kDa) show endogenous Akt and upper bands (87 kDa) depict eGFP-fused Akt1-mutants. (**B**) The quantification of pMERIT40 western blots of 3 independent experiments shows volume intensity normalized to the background. Volume intensity of phosphorylated Akt was normalized to the volume intensity of the total amount of Akt. Bars represent means ± SD from 3 independent experiments. * *p* < 0.05, ** *p* < 0.01, **** *p* < 0.0001; ANOVA test with Tukey correction.

We thus wondered if the genetic or pharmacologic inhibition of Akt would be associated with reduced phosphorylation of target proteins involved in the regulation of DSB repair. Here, we focused on MERIT40, a documented Akt target protein involved in HRR [14,15,21]. As shown in Figure S3A, the overexpression of the phosphorylation-deficient Akt1 mutants showed decreased phosphorylation of MERIT40 before and after IR compared to cells overexpressing Akt1-WT (Figure S3A). Herein, the overexpression of Akt1-TASA was more effective in reducing MERIT40 phosphorylation than the single phosphorylation-deficient mutants, at least in the irradiated cells (Figure S3A). Of note, the genetic inhibition of Akt1 in the double-phosphorylation-deficient Akt1-TASA mutant was almost similarly effective in inhibiting MERIT40 phosphorylation in irradiated cells as pharmacologic inhibition of Akt-activity in Akt1-WT overexpressing TrC1 by pre-treatment of these cells with MK-2206 (Figure 5A,B; Figure S3A). We speculate that the slightly enhanced ability of MK-2206 to inhibit MERIT40 phosphorylation in irradiated TrC1 when compared to the overexpression of Akt1-TASA might be due to its ability to inhibit also the effects of the intact endogenous Akt, that is highly activated in the Akt1-WT and Akt1-TASA cells upon irradiation (Figure 5A). To evaluate the potential effects of intact endogenous Akt1, we additionally tested the phosphorylation of MERIT40 in our recently described Akt1-deficient (Akt^−/−^) murine embryonic fibroblasts (MEFs) overexpressing the dominant negative Akt1-K179A-mutant [7]. In support of the above assumption, we failed to detect a phosphorylation of MERIT40 in the Akt1-K179A overexpressing Akt1^−/−^ MEFs without and with irradiation by using Western Blot analysis suggesting that active Akt—either endogenous or overexpressed—is required for MERIT40 phosphorylation (Figure S3B). 

To further explore the suggested link between the Akt activation state and the phosphorylation of MERIT40, we additionally investigated the impact of the overexpression of the activation-associated and clinically relevant Akt1-E17K mutant described in our recent publication [7]. Of note, here we observed a strong increase in radiation-induced phosphorylation of MERIT40 in Akt1-E17K overexpressing TrC1 (Figure 5A,B). 

Taken together, the phosphorylation-deficient mutants have a reduced ability to phosphorylate MERIT40, a documented downstream effector protein of Akt involved in DSB repair. Though the relative roles of the expression versus phosphorylation state of MERIT40 for the repair of radiation-induced DSB remains to be explored, it is highly likely that the protein phosphorylation will impact protein localization, protein stability, or its action as a scaffolding protein for other DSB repair proteins, respectively. 

We, therefore, used the Kaplan-Meyer-Plot tool to explore a potential link between MERIT40 expression and the survival of cancer patients using publicly available databases [22,23]. Though no data on prostate cancer were available, these analyses revealed an association of high MERIT40 expression with reduced progression-free or overall survival in cancer patient samples from ovaries and gastric tract, respectively [22,23] (Figure S3D). Of note, the higher expression of MERIT40 and poorer overall survival correlated with higher expression of Akt1 particularly in gastric cancer patients (Figure S3C,D).

## 3. Discussion

Akt is an important survival kinase with clinical relevance to radiation resistance. Here, we reveal that DNA-PKcs functions as an upstream kinase of IR-mediated Akt phosphorylation at S473 in intact cells using DNA-PKcs-deficient and proficient glioblastoma cells. Moreover, we demonstrate that the overexpression of the Akt1-TASA mutant that is deficient in phosphorylation of Akt’s two major activation-associated phosphorylation sites, T308 and S473, decelerated the repair of radiation-induced DSB and increased radiosensitivity of TrC1 prostate cancer cells when compared to Akt1-WT overexpressing TrC1. This implicates the Akt’s activation state in the cellular response to IR and DSB repair. However, the phosphorylation state was not important for the ability of Akt to gain nuclear access. 

In more detail, several published reports suggested that mTORC2 or DNA-PKcs function as upstream kinases phosphorylating Akt1 S473 in response to growth factor stimulation or DNA damage, respectively [6,24,25,26] (reviewed by Reference [5]). Our own previous findings gained from an in vitro kinase assay demonstrated that DNA-PKcs indeed functions as an upstream kinase activating Akt1 at S473 in the presence of damaged DNA and not vice-versa [7] as suggested in other studies [27]. In the present study, we confirmed our previous observations in an intact cellular system by showing that DNA-PKcs-deficient M059J glioblastoma cells failed to induce the S473 phosphorylation upon IR, whereas DNA-PKcs-proficient M059K cells showed a significant but transient increase of phosphorylation at S473 at 30 min after IR. This is consistent with our earlier findings that the overexpression of activation-associated Akt1 mutants Akt1-E17K and Akt1-TDSD accelerated the repair of radiation-induced DSB particularly between 2 h and 6 h after irradiation [7]. Herein, the ability of DNA-PKcs to phosphorylate Akt at S473, presumably in the nuclear compartment, might allow cells without genetically-induced aberrant Akt-activation to enhance DSB repair by phosphorylating downstream nuclear targets involved in the repair of radiation-induced DNA damage [6,7,24] (reviewed by Reference [5]).

Instead, the increased radiosensitivity of the Akt1-TASA overexpressing cells revealed in the present study was associated with deceleration of DSB repair upon irradiation and reduced phosphorylation of Akt target proteins such as FOXO1 with reported importance to the regulation of cell survival (FOXO-transcription factors) [18]. While blocking only one of the two major phosphorylation sites of Akt (T308 or S473) still allowed the cells to undergo normal FOXO1-phosphorylation, genetic inhibition of both phosphorylation sites in Akt1-TASA overexpressing cells resulted in reduced FOXO1 phosphorylation and was associated with a strong inhibitory effect on the long-term survival of irradiated cancer cells. The observation that similar effects could be achieved by pre-treating Akt1-WT overexpressing TrC1 with MK-2206 suggests that the negative regulation of FOXO-proteins with a documented role in the regulation of cell-cycle arrest, apoptosis, the DNA damage response and resistance against oxidative stress [28] (reviewed by Reference [29]) might contribute to the increased radiosensitivity of Akt1-TASA overexpressing TrC1.

In an attempt to gain further insight into the underlying mechanisms, we could additionally correlate increased radiosensitivity of Akt1-TASA overexpressing TrC1 with a significant delay in the resolution of radiation-induced γH2A.X foci indicative of DSB. These findings were corroborated by a delay in the repair of radiation-induced DSB in Akt1-TASA overexpressing TrC1 using the neutral comet assay. Again, the overexpression of the phosphorylation-deficient Akt1-TASA mutant and pre-treatment of Akt1-WT overexpressing cells with the Akt-inhibitor MK-2206 had similar inhibitory effects on DSB repair, whereas the effects of the single phosphorylation mutant Akt1-SA were less pronounced. This suggests that the S473 phosphorylation might be more important for the activation of downstream effector proteins with a relevance to the repair of radiation-induced DSB, possibly by altering the spectrum of effector proteins [30,31,32]. 

In contrast, the repair of radiation-induced DSB in Akt1-TA overexpressing cells was similar to Akt1-WT overexpressing cells. In this context, it might be important that Akt1-SA expressing cells were still able to undergo phosphorylation at T308 whereas TrC1 overexpressing Akt1-TA failed to undergo phosphorylation at S473. The observation that T308 phosphorylation seems to be required for subsequent phosphorylation at S473 in the cell system used in our study corroborates earlier findings about the importance of T308 phosphorylation to unfold Akt-activity and allow for the complete activation of Akt by additional phosphorylation at S473 under various conditions [33] (reviewed by [4,5]). However, to prove the suggested hierarchical role of T308 and S473, this observation should be confirmed in an Akt1-deficient cellular system.

Pronounced phosphorylation and thus, the activation of Akt, was required for improving DSB repair and long-term survival upon exposure to IR in our earlier study and this was associated with the increased nuclear localization of the activation-associated and resistance-promoting Akt1-mutants [7]. In line with these findings, earlier reports suggested that active Akt1 rapidly translocates to the nucleus upon IR [7,34,35]. However, it remained controversial if cytoplasmic phosphorylation of Akt is dispensable for its nuclear access: Nguyen and colleagues showed that Akt1-TA and Akt1-SA failed to translocate to the nucleus in PC12 cells [36], whereas the double phosphorylation-deficient mutant Akt-TASA was found in the nuclear compartment using live imaging in hepatocellular carcinoma cells (HCC) [37]. Here, we now demonstrate that the overexpressed eGFP-Akt1 mutant proteins could localize to the nuclear compartment of TrC1 prostate cancer cells under basal conditions independently of their ability to undergo phosphorylation. Moreover, treatment of Akt1-WT expressing TrC1 with the allosteric Akt-inhibitor MK-2206 did not reduce basal nuclear Akt1-levels. Finally, the exposure to IR induced a comparable slight increase in nuclear Akt1-eGFP in all tested Akt1-mutants. However, the overexpression of Akt1-SA or Akt1-TASA as well as the pre-treatment of Akt1-WT expressing TrC1 with the allosteric Akt-inhibitor MK-2206 all reduced the kinetics of DSB repair and increased the sensitivity of TrC1 to IR when compared to cells overexpressing Akt1-WT. These observations implicate that (i) phosphorylation at S473 and T308 is not required for Akt1 to gain nuclear access, and (ii) that simple accumulation of Akt in the nuclear compartment is not sufficient to promote DSB repair and radioresistance. Instead, we propose that the presence of phosphorylated active Akt is required to improve DNA repair and increase radioresistance. Instead, it is not required that Akt undergoes phosphorylation before translocating to the nucleus. 

Earlier reports proposed that active Akt might influence DSB repair through phosphorylating targets involved in NHEJ such as XLF or UBE2S [9,10] or HRR, e.g., by phosphorylating MERIT40 [15]. It has been described that MERIT40 cooperates with BRCA2 (breast cancer 2) to resolve DNA interstrand cross-links [38]. Moreover, MERIT40 has been linked to HRR through the interaction with ABRAXAS bridging molecule to generate a scaffold among various members of the BRCA1-complex and further stabilize the entire complex [21]. Thereby, MERIT40 controls and improves the integrity of the BRCA1-complex and its accumulation at the DNA damage site [14,21] to facilitate DSB repair [39]. In that study, the authors proposed that the scaffolding action occurs independently of MERIT40-phosphorylation [21]. However, there is some evidence that the phosphorylation of MERIT40—induced for example by treatment with the chemotherapeutic drug doxorubicin—might facilitate the assembly of the BRCA1 complex at the DNA damage site thereby improving repair of DSB [15]. Instead, the abrogation of Akt-mediated MERIT40-phosphorylation impaired the assembly of the BRCA1 complex at DNA damage sites leading to decelerated DSB repair and reduced cell survival following the doxorubicin treatment [15]. 

In this context, we find it intriguing that increased radioresistance of Akt1-E17K overexpressing TrC1 was associated with the increased phosphorylation of the Akt target protein MERIT40 whereas the increased radiosensitivity of Akt1-TASA overexpressing cells was associated with a reduced MERIT40 phosphorylation. Moreover, the enhanced phosphorylation of MERIT40 in Akt1-E17K expressing cells correlated with significantly accelerated DNA repair compared to Akt1-WT expressing cells, whereas, in Akt1-TASA overexpressing cells or in Akt1-WT cells treated with the MK-2206 inhibitor, the repair of IR-induced DSB was impaired. It is therefore tempting to speculate that impaired phosphorylation of MERIT40 in cells with genetic or pharmacologic inhibition of Akt might contribute to some extent to decelerated DSB repair, radiosensitivity, or both. 

However, MERIT40 is only one of potential Akt targets that may influence DSB repair processes and survival upon DNA damage. Thus, further work is required to dissect the role of nuclear Akt effector proteins in the repair of radiation-induced DSB repair and this will be subject to future studies.

## 4. Materials and Methods

### 4.1. Chemicals, Antibodies, and Drugs

The MK-2206 Akt-inhibitor was purchased from Selleckchem (Houston, TX, USA). The mouse monoclonal ß-Actin antibody was obtained from Sigma-Aldrich (St. Louis, MO, USA). panAkt, pS473, pT308, panMERIT40, pMERIT40, pFOXO1/3a, panFOXO1, eGFP and ß-Actin antibodies were purchased from Cell Signaling Technology (Danvers, MA, USA). Alexa Fluor 647-coupled antibody against γH2A.X protein was obtained from Becton Dickinson (Heidelberg, Germany). Hoechst33342 was purchased from Invitrogen (Eugene, OR, USA). DAKO Fluorescent mounting medium from Dako North America Inc. (Carpinteria, CA, USA) was used. All other chemicals were acquired from Sigma-Aldrich (St. Louis, MO, USA).

### 4.2. Cell Culture, Drug Treatment, and Irradiation

M059J (DNA-PK deficient) and M059K (DNA-PK proficient) human glioblastoma cell lines [16] were kindly provided by Prof. George Iliakis (Institute of Medical Radiation Biology, UK Essen, Germany) and cultured in DMEM supplied with 10% (*v*/*v*) fetal calf serum. The Platinum-E (Plat-E) cell line and TrC1 murine prostate adenocarcinoma cells were purchased from the ATCC (Bethesda, MD, USA). Murine embryonic fibroblasts (MEF) with an Akt1 knock-out (Akt1^−/−^) were kindly provided by Morris J. Birnbaum (Philadelphia, PA, USA). TrC1 were cultured in DMEM (Life Technologies, Darmstadt, Germany) medium supplemented with 10% (*v*/*v*) fetal calf serum (Biochrom, Berlin, Germany) and maintained in a humidified CO_2_ incubator at 37 °C and 5% CO_2_ (Labotect, Goettingen, Germany). Transduced TrC1 were cultured two weeks before and after eGFP-based cell sorting in a DMEM medium containing 10% FCS, 1 × Pen/Strep, and 4 µg/mL Puromycin (selection and contamination prevention). For radiation treatment, the cells were exposed to 3 Gy using an X-RAD 320 X-Ray Biological Irradiator with a MIR-324 X-ray tube at a dose rate of 3.6 Gy/min and analyzed at two hours after irradiation (Precision X-Ray Inc., North Branford, CT, USA). The dosimetry was performed by the physicists of the Department of Medical Radiobiology, Essen, Germany. Pretreatment with the inhibitor MK-2206 (purchased from Selleckchem, Houston, TX, USA) diluted in culture medium was performed at indicated times before irradiation.

### 4.3. Generating Stably Akt1 Mutant Expressing Cells

We used the ecotropic retroviral expression system for the virus production. The expression plasmid pBABE with a puromycin resistance contained human Akt1-WT, -T308A, -S473A and T308A/S473A mutants. Plat-E cells were seeded and transfected with a plasmid using the TransIT-LT1 transfection reagent when 50–70% of confluency was reached. Plat-E cells release viruses into the medium immediately upon transfection. Medium containing viruses with each Akt1 construct was filtered using a 0.45 µm filter before added to the target murine cell line TrC1. To increase the transduction efficiency, we added 4 µg/mL of hexadimethrine bromide. After 24 h of incubation at 37 °C, the transduced TrC1 were selected by 1 µg/mL puromycin. After 24 h, the concentration of puromycin was increased to 4 µg/mL.

### 4.4. Immunofluorescence Staining

The cells were fixed and permeabilized using 3% para-Formaldehyde (PFA) and 0.2% Triton X-100 in PBS; 15 min; RT, respectively. After washing with PBS, the cells were blocked overnight with 2% goat serum in PBS. The antibodies were diluted in blocking buffer. Alexa Fluor 647-conjugated anti-γH2A.X antibody was incubated for 1 h at a 1:100 dilution. The samples were washed after each incubation step three times with PBS followed by staining for 15 min in the dark with 0.2% (*w*/*v*) Hoechst33342 in PBS. The samples were again washed with PBS, mounted with the DAKO mounting medium and stored at 4 °C in the dark. Single layer fluorescence images were taken with a Zeiss AxioCam MRm (1388 × 1040 pixels) at a Zeiss Axio Observer Z1 fluorescence microscope with Plan-Apochromat 63 × /1.40 Oil M27 lens, 49 DAPI, 38 HE, 43HE and 78 HE ms filter and a transmission grid VH “ApoTome” (Carl Zeiss, Goettingen, Germany). Images were taken with three fourth of the maximum intensity without overexposure. The pictures were saved as 16-bit multi-channel Carl Zeiss Image files (CZI) with no further editing. γH2.AX-foci counted with the Focinator software as previously described [20], eGFP integrated intensity was performed by CellProfiler [19].

### 4.5. Colony Formation Assay

Clonogenic cell survival was tested in response to radiotherapy with doses between 1 and 10 Gy. Exponentially growing cells were seeded in 6-well plates and irradiated 24 h later. For the determination of colony formation, the cells were fixed in 3.7% formaldehyde and 70% ethanol, stained with 0.05% Coomassie blue. Colonies of at least 50 cells were counted.

### 4.6. Comet Assay

Comet assay was performed using a modified protocol of Olive and Banath [40]. Glass slides were pre-coated with 1% agarose. Cells were seeded in a 6-well plate, irradiated 24 h later with 40 Gy and collected at the indicated time points by trypsinization. Cells were gently resuspended in 120 μL of 1% 2-Hydroxyethylagarose from Sigma-Aldrich (St. Louis, MO, USA), directly pipetted onto the coated glass slides and immediately covered by a cover slip. Neutral lysis was performed in N1 buffer (2% Sarkosyl, 0.5 M Na_2_EDTA, 0.5 mg/mL proteinase K (pH 8.0)) for 3 h at 37 °C 5% CO_2_ and stopped with N_2_ buffer (90 mM Tris buffer, 90 mM boric acid, 2 mM Na_2_EDTA (pH 8.5)) for 15 min. Electrophoresis was conducted at 15 V (0.6 V/cm) for 25 min. The comets were stained with 50 μg/mL propidium iodide. For analysis, at least 50 comets were examined for their tail area using the software OpenComet.

### 4.7. Flow Cytometry

Flow cytometry analysis of the cell cycle distribution was based on propidium iodide (PI) staining of the DNA in a hypotonic citrate buffer as described earlier [7]. In brief, cells were resuspended in 100 μL of the staining solution (comprised of 0.05% Triton X-100, 50 μg/mL propidium iodide and 0.1% sodium citrate in PBS), incubated in the dark for 30 min and measured with a BD 21 Accuri C6 flow cytometer (BD Biosciences, San Jose, CA, USA). Flow cytometry analyses were performed using BD Accuri C6 Software (BD Biosciences).

### 4.8. Statistical Analysis

The data represent mean values of at least 3 independent experiments ± standard deviation (SD). The data analysis was performed by t-test or two-way ANOVA test with Tukey comparison post-test and determination coefficient calculation where appropriate using Prism6TM software (GraphPad Inc., La Jolla, CA, USA). *p* values ≤ 0.05 were considered as significant.

## Supplementary Materials

Supplementary materials can be found at http://www.mdpi.com/1422-0067/19/8/2233/s1.
