# Supplementary material for: Restraining Akt1 Phosphorylation Attenuates the Repair of Radiation-Induced DNA Double-Strand Breaks and Reduces the Survival of Irradiated Cancer Cells
Source: Int J Mol Sci. 2018 Jul 31;19(8):2233. doi: 10.3390/ijms19082233 (PMC6121313; doi:10.3390/ijms19082233)
Supplement: Supplementary file 1 [file ijms-19-02233-s001.pdf]

Supplementary materials

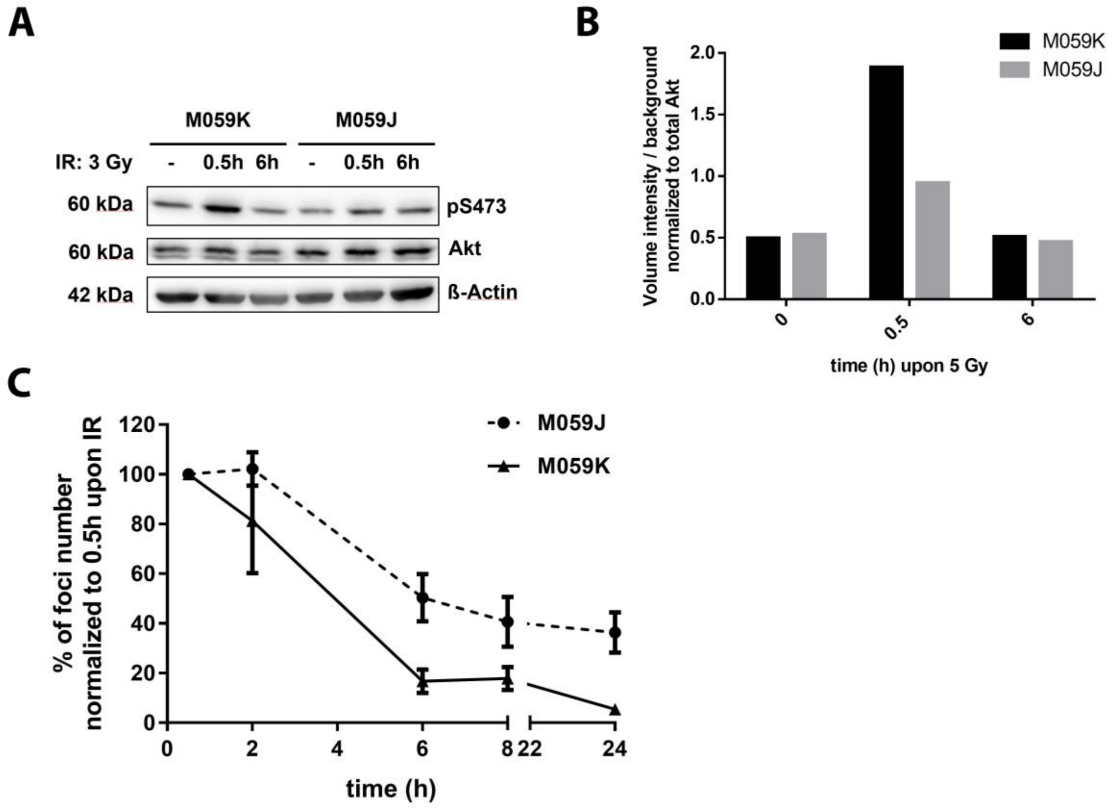

Figure S1. Genetic inhibition of DNA-PKcs affects DNA repair upon IR. (**A**) Human glioblastoma cell lines M059J (DNA-PK-deficient) and M059K (DNA-PK-proficient) were exposed to irradiation with 3 Gy. Phosphorylation (pS473) and expression status of Akt were analyzed by Western blot analysis 0.5 and 6 h upon IR and in non-irradiated (-) cells. (**B**) Bars depict quantification of volume intensity of pS473 in Western blots in 1 out of 3 independent experiments normalized to background. Volume intensity of phosphorylated Akt-S473 was normalized to volume intensity of total amount of Akt. (**C**) M059J and M059K cells were exposed to irradiation with 3 Gy and fixed in 3% PFA, permeabilized with 0.2% Triton X-100 in PBS at distinct time points between 0 h and 24 h upon irradiation and stained with Hoechst33342 and γH2A.X. Data show quantification of γH2A.X foci at 0.5-24h after irradiation with 3 Gy by using the Focinator v 2.2 software [20]. Values were normalized to amount of foci detected at the 0.5 h time point.

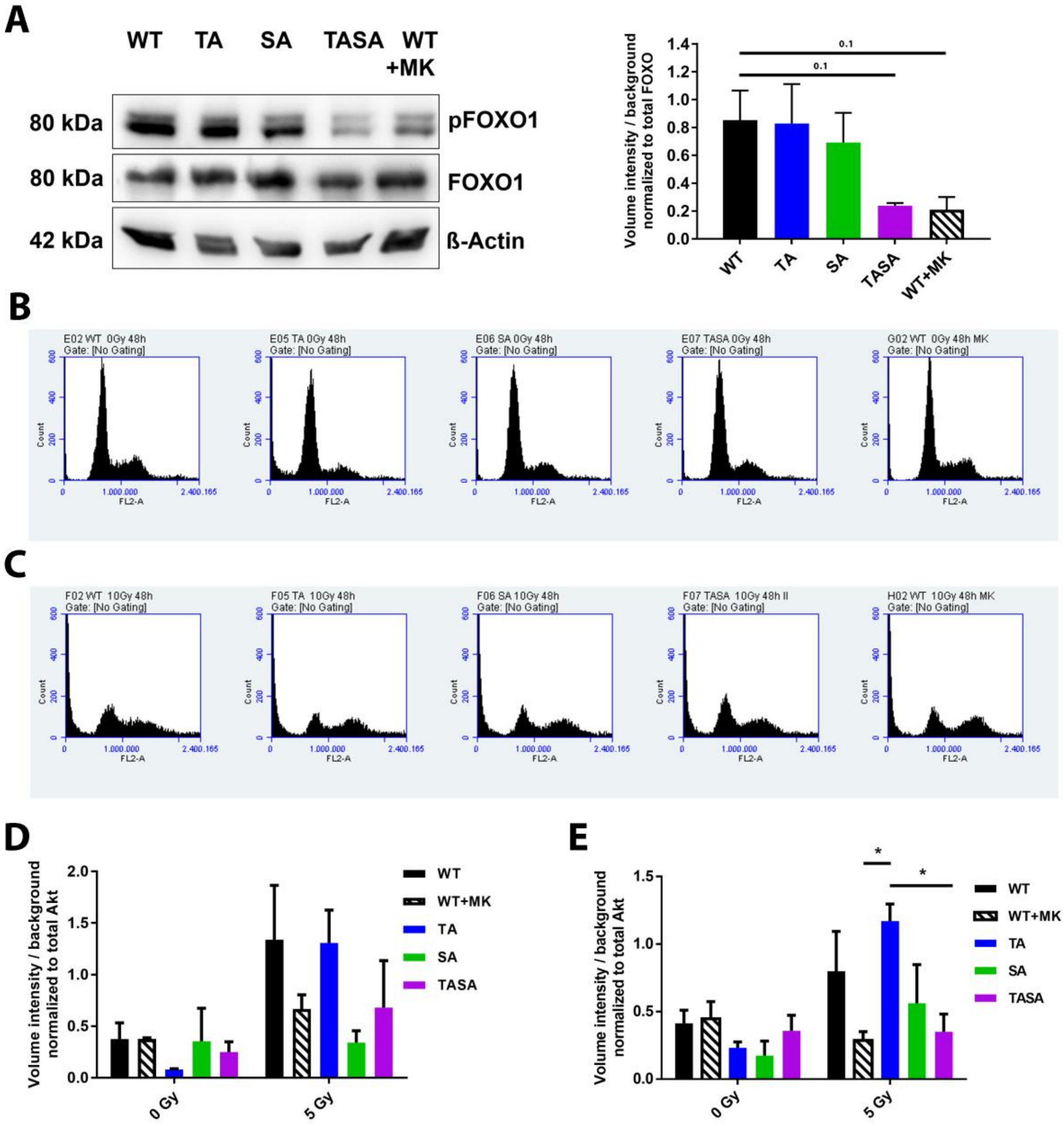

Figure S2. (**A**) Basal phosphorylation status of FOXO1 in all depicted Akt1 mutants stably expressed in TrC1 analyzed by western blot. Akt1-WT expressing cells were treated with MK-2206 inhibitor (4 µM) 16 h before IR. Quantification of pFOXO1 western blots shows volume intensity normalized to background performed by Phoretix software. Volume intensity of phosphorylated FOXO1 was normalized to volume intensity of total amount of FOXO1. Bars represent mean values ± SD from 3 independent experiments. Significances were tested with student's t-test. (**B**,**C**) Exemplary plots of cell cycle distribution in non-irradiated (**B**) and with 10 Gy irradiated (**C**) Akt1-WT, Akt1-TA, Akt1-SA, Akt1-TASA mutants and Akt1-WT cells treated with MK-2206 inhibitor (4 µM; 16 h preincubation) analyzed by flow cytometry after 48 h incubation. Quantification of (**D**) pS473 (endogenous Akt) and (**E**) pT308 (endogenous Akt) Western blots shown in Fig. 2 A. Bars show volume intensity normalized to background performed by Phoretix software. Volume intensity of phosphorylated S473 or T308 was normalized to volume intensity of total amount of Akt. Bars represent mean values ± SD from 3 independent experiments. ANOVA test with Tukey correction. * $p<0.05$.

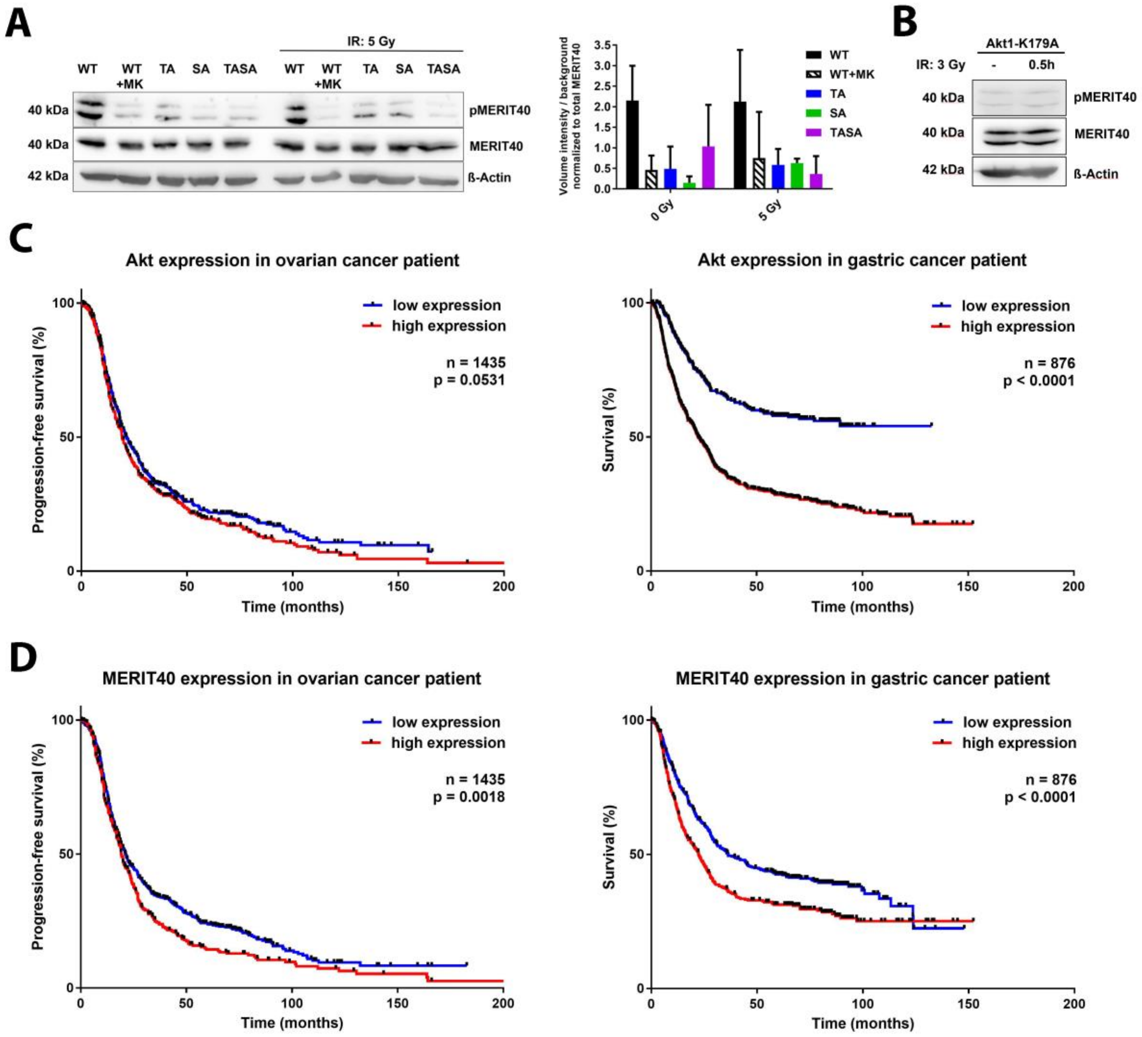

Figure S3. (**A**) Phosphorylation status of MERIT40 in TrC1 cells stably expressing different Akt1 mutants revealed by western blot analysis. Akt1-WT expressing cells were treated with MK-2206 inhibitor (4 µM) 16 h before IR (WT + MK). Quantification of pMERIT40 western blots shows volume intensity normalized to background performed by Phoretix software (right panel). Volume intensity of phosphorylated MERIT40 was normalized to volume intensity of total amount of MERIT40. Bars represent mean values ± SD from 3 independent experiments. (**B**) Phosphorylation status of MERIT40 in Akt1-K179A mutant stably expressed in MEF Akt1-/- cells tested by western blot. (**C**,**D**) Kaplan-Meier-Plots of ovarian cancer (left panels) and gastric cancer patients (right panels) performed in a cohort of 1435 and 876, respectively. Plots represent time-dependent overall survival. (**C**) High Akt1 expression in gastric cancer patients (right) correlates with significantly reduced overall survival ($p<0.05$). (**D**) High MERIT40 expression in ovarian (left) and gastric cancer patients (right) correlates with reduced overall survival.
